# Unpacking the emotional drive: a grounded theory model of online collective action in social media

**DOI:** 10.3389/fpsyg.2026.1753234

**Published:** 2026-03-02

**Authors:** Shuang Li, Jiajia Hao, Tianyi Li, Yixuan Zhang, Tongyue Feng, Xiaoxia Zhu

**Affiliations:** 1 Economics Management School, Yanshan University, Qinhuangdao, Hebei, China; 2 Institute for Advanced Studies in Humanities and Social Sciences, Beijing Normal University, Zhuhai, Guangdong, China

**Keywords:** emotional contagion, grounded theory, online collective action, relative deprivation, social identity, trust dynamics

## Abstract

What are the underlying psychological motivations that drive online collective action? This study addresses this question by employing a constructivist grounded theory methodology to analyze over 300,000 words of textual data from 16 influential cases on Chinese social media. This study operationalizes “emotional drive” as the core dynamic process in online collective action wherein group emotions, triggered by an initial event and rapidly contagious and amplified via social media, interact with group social identity and external action constraints to nonlinearly propel collective action from emergence and diffusion to transformation. We developed a comprehensive theoretical model that identifies “emotional drive” as the core phenomenon, around which five primary categories revolve: emotional contagion, trust dynamics, relative deprivation, social identity, and action constraints. Our findings reveal that emotional contagion acts as the central catalyst, often sparked by a pervasive sense of relative deprivation among participants. This research provides a nuanced social-psychological framework for understanding how large-scale collective behaviors emerge from the interplay of individual cognition and group dynamics in digital environments. The model not only offers theoretical insights but also yields practical implications for the governance of online communities and the management of social dynamics in the digital age.

## 1 Introduction

In today's world of rapid technological advancement, the organizational forms and behavioral logic of contemporary society are being increasingly and profoundly shaped by Internet technology (Liu et al., 2021), which also makes channels for citizens to participate in politics and governance more accessible (Zheng and Chen, 2024; Yu, 2023). Online collective action can quickly assemble a large number of netizens, forming powerful public opinion pressure and action capabilities, and exerting a profound impact on social issues (Wu and Xiao, 2024). Adopting online collective action, netizens can express common demands and wishes, promoting changes. Traditional methods of civic participation into discussing social issues were often limited by geographical, temporal, and other factors, resulting in generally limited influence. However, online collective action can break these limitations, providing feasibility for more people to participate in social affairs (Li, 2024). By using online platforms, people can more conveniently access information, express opinions, and organize actions, enhancing the efficiency and breadth of civic participation. Netizens also conduct democratic supervision over government departments, enterprises, and other organizations through public opinion pressure by exposing scandals, and other means. This form of supervision has characteristics such as anonymity, broad reach, and immediacy, enabling it to quickly expose problems, attract attention, and promote problem resolution.

Actually, with the intensive usage of the Internet, the virtual world built online has essentially become the “primary dimension” for community existence and activity; therefore, the organizational forms and action logic of online activities will permeate into real life (Jin, 2024). When social problems arise, online collective action can timely accumulate the strength of netizens, reinforce public opinion pressure, and promote the resolution of problems. In the Sun Haiyang child-searching incident, netizens made positive contributions to finding Sun Zhu by actively providing clues, launching fundraisers, calling for attention and help, and spreading positive energy and warmth. These actions not only made Sun Haiyang and his wife feel the care and support of society but also raised public awareness and understanding of the issue of child trafficking. From this perspective, online collective action can sometimes enhance social cohesion and promote social harmony and stability. Therefore, in the face of novel and complex online collective action emergencies, analyzing their formation drivers and summarizing their evolutionary patterns is conducive to better understanding and responding to this social phenomenon, more efficiently understanding the various demands society puts forward, and better coping with opportunities and challenges in the broader context of social transformation.

## 2 Research design

### 2.1 Literature review

Emotional contagion, as the initial impetus for online collective action, activates group behavior through many digital communication pathways. It activates group behavior through an automated chain of “unconscious mimicry-emotional arousal-behavioral diffusion” (Zhang and Lu, 2013). Consensus mobilization on social platforms triggers emotional arousal, and individual emotional sensitivity positively moderates this process (Zhang, 2025); positive emotional information enhances sharing intentions through direct or mediating pathways (Shi et al., 2025), while negative emotions like panic spread via an “epidemic model,” where lower emotional thresholds lead to larger infection scales (Huo et al., 2023; Dong et al., 2023). This contagion exhibits significant asymmetry. For example, in e-commerce promotions, the contagious power of anticipated joy far exceeds that of regret in purchasing goods (Lu, 2023), thus resulting in carnival-style collective consumption. Relying on data from the China Social Survey (CSS), the complete mediating function of social perception between interest satisfaction and social identity is demonstrated (Wu and Ding, 2024), indicating that enhancing the sense of social fairness, security, and tolerance among the emerging youth group is pivotal to strengthening their identity and reducing confrontational collective action. Furthermore, research has further revealed the core roles of emotional contagion and relative deprivation in online collective action. Studies show Lilly et al. that their dynamics and group differences significantly impact collective behavior: using multi-trajectory latent class growth analysis, long-term change trajectories of relative deprivation were identified (Lilly et al., 2025), revealing how it, as a psychological representation of social inequality, drives group mobilization.

Identity is able to form cohesion, constructing a “we” group boundary and excluding the “other” from the group. Identity and emotional contagion do not act in isolation but are intertwined and influenced, jointly promoting the generation and evolution of online collective action. Research on empathic communication in e-sports events found that emotional contagion undergoes three stages: “emotional infection—perspective-taking—empathic concern” (Wang and Li, 2025), ultimately triggering altruistic behavior and emotional projection in the audience. Besides, this mechanism is also applicable to explaining emotional mobilization in online collective action. However, emotional mobilization mainly relies on “underdog narratives” and “media boosts” (Ding, 2022), achieving rapid emotional aggregation and dissemination by activating the public's negative social mentality and cultural sentiments (such as chivalry). For instance, online patriotism stimulates confrontational emotional logic through national identity (Liu, 2021), while standup comedies reconstruct marginal identity through self-deprecation (Zhou, 2025). The “swarm effect” further explains how dispersed individuals can form a group consciousness with common goals through emotional resonance (Zhang X. J. et al., 2025), pushing collective action toward extremism. In recent years, research on collective action has gradually shifted from a single-emotion driver to an “emotion-reason” dual-pathway model (Shi et al., 2023). Procedural injustice predicts online collective action intentions through emotion-focused coping, while action support functions through both emotion- and problem-focused coping pathways (Shi et al., 2023), providing a framework for understanding the multi-dimensional motivations of online mobilization. Additionally, research on group relative deprivation has extended to extreme behaviors. In French Muslim communities, group relative deprivation was found to indirectly predict violent radicalism through activism, and group contempt uniquely predicted support for violence (Guimond et al., 2025), revealing the emotional mechanisms by which deprivation transforms into extreme action.

In the current context of deep integration between digitalization and social media, online collective action has become an important form of social movement and public expression. In public crises, technological empowerment promotes sustainable collective participation by enhancing efficiency, like instrumental rationality and stimulating a sense of responsibility, such as social identity (He et al., 2024). However, forced identification through didactic discourse can trigger rejection, undermining action consensus (Li R. et al., 2025). Furthermore, a sense of relative deprivation and anger rumination play a chain-mediating role between belief in a just world and malicious creativity (Xiao et al., 2024), suggesting that negative emotions and identity threats may exacerbate irrational collective behavior. Therefore, understanding online collective action requires a foundation in the synergistic effects of emotion and identity. It is necessary to focus on the identity construction and emotional resonance within the group, without ignoring the shaping and amplification effects of the external social environment and technological platforms. Online self-organized actions, like the “Lei-Huo Volunteers,” form distributed collaboration through value-driven, relationship-driven, and technology-driven mechanisms, completing information fusion and resource dispatch (Yu and Zhang, 2022). However, algorithmic recommendations lead to information stratification (Yan, 2024), which, while strengthening intra-group identity, exacerbates “information cocoons” and inhibits cross-group mobilization capabilities. From the perspective of “high-quality development,” it is emphasized that online emotional governance needs to start from the psychological generation mechanisms and communication elements of its subjects, to strengthen monitoring and promote the construction of a civilized online ecosystem (Guo and Lin, 2023). From the perspective of national security, a framework for multimodal language public opinion monitoring points out that in the “post-truth” era, focus should be on online emotions and mainstream ideological security, incorporating multimodal symbols (such as deepfakes, emojis) to accurately identify negative emotions and ideological threats (Pan, 2024). Therefore, it is not difficult to understand that the essence of online collective action is a ternary aggregation of emotion, identity, and technology. An analysis of the driving factors of online collective action will provide governance insights for guiding online creativity.

### 2.2 The theoretical context and innovative positioning of “emotional driving force”

More recently, studies have revealed how emotion reshapes the forms and relationships of news production and consumption (Wang C. Z., 2023), and directly influences user creation and dissemination behaviors through social platform algorithms (Yang Y. Q. et al., 2023). Even generative artificial intelligence, represented by ChatGPT, deeply intervenes in the shaping of public-political discourse by constructing new patterns of interactive emotion (Zhang and Jia, 2023). In public crisis management, emotional analysis has become a crucial basis for measuring the effectiveness of mainstream media discourse guidance and implementing precise emotional governance (Zhang and Wei, 2021).

These studies provides a solid, cross-disciplinary academic consensus for positioning “emotion” at the core of the motivational model for online collective action in this study. Most existing research, however, operates within a single analytical layer–focusing either on micro-level individual psychology and behavior, meso-level organizational and industry transformations, or macro-level discursive atmospheres and strategies. In contrast, this study aims to integrate micro, meso, and macro perspectives to construct a cross-level dynamic model that connects individual emotional contagion, group social identity, and platform/societal action constraints. Its goal is to systematically unveil the complete chain from emotional triggers to the formation of collective action. The methodologies employed in the literature range from theoretical speculation and case studies to big data emotional computing and quantitative modeling. The constructivist grounded theory method adopted by this study addresses a gap among these approaches: it is capable of both in-depth theoretical construction based on rich qualitative data and capturing complex, dynamic group interaction processes, making it particularly suitable for explaining process-heavy, highly contextual phenomena like online collective action.

Therefore, building upon the existing scholarly consensus regarding the core driving value of emotion, this study focuses on the specific and complex phenomenon of online collective action. Utilizing the grounded theory method, it seeks to construct an integrated, multi-faceted process model of “emotional driving force.” This model not only represents a concretization and contextualization of emotional mobilization theory within the domain of online collective action but also attempts to supplement and integrate existing theories by revealing how emotion interacts with identity construction, technological affordances, and institutional constraints to drive action. Its innovation lies in providing a systematic middle-range theoretical framework for understanding the generative mechanisms of non-institutionalized, spontaneous collective action in the digital age.

In summary, the concept of “emotional drive” proposed in this study specifically refers to an integrative analytical framework. It does not denote a singular phenomenon of “emotion” or “emotional contagion,” but rather emphasizes emotion as a core dynamic—a key mechanism that drives the entire process of online collective action through its dynamic interaction with structural and contextual factors such as “social identity” and “action constraints.” This distinguishes it from traditional collective action theories internationally, which tend to focus on resource mobilization, political processes, or frame alignment. The innovation of this model lies in the fact that it not only transcends the traditional perspective that treats emotion as a subsidiary to rationality or a static background element but also addresses the inadequacy of single-mechanism emotional approaches in explaining action transformation and constraints. Through qualitative analysis grounded in the Chinese context, this model reveals how emotion, as a bottom-up, fluid “driving force” within a digitalized and decentralized communication ecosystem, systematically bridges micro-level psychology, meso-level groups, and macro-level techno-institutional environments. Thereby, it provides a more dynamic and integrative middle-range explanatory framework for understanding non-institutionalized, flash-mob-style online collective actions.

### 2.3 Research methodology

The causes and catalysts of online collective action are often varied and diverse. First, participants usually have differences in all aspects, such as different life backgrounds, internet preferences, and ways of thinking. How to observe and summarize consistency among numerous differences has become an urgent topic to be resolved. Second, by understanding the widely impactful cases that have occurred on China's Internet in recent years, it is not difficult to find that the public voices contained within them have observational value for forming theories and practical value for improving citizen participation channels. Finally, whether the observed influencing factors are sufficiently generalizable and abstract is one of the evaluation criteria for the completeness of the research. Therefore, adopting the grounded theory method for refinement and induction can be considered.

In the field of qualitative research, a highly renowned method is “Grounded Theory” (Glaser et al., 1968). Grounded theory places particular emphasis on developing theory from data, arguing that a theoretical framework can only be gradually formed through in-depth analysis of the data. It is an inductive process that continuously condenses data from the bottom up (Chen, 1999). Scholars have also traced the development of grounded theory and its different schools, comparing the data coding processes of the most representative classical grounded theory, procedural grounded theory, and constructivist grounded theory, and outlining their evolution and pathways (Jia and Heng, 2020).

**Figure 1:**
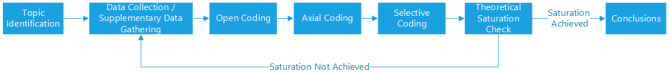
Grounded theory flowchart.

The choice of this method is mainly based on the following three considerations: First, the driving factors of online collective action are complex, diverse, and intertwined, and participants come from varied backgrounds. Traditional quantitative questionnaires struggle to capture their deep, dynamic evolutionary mechanisms. Second, this study aims to generate theory from empirical data from the bottom up, rather than verifying existing hypotheses. The core principle of grounded theory, “constructing theory from data,” is highly consistent with this goal. Third, this method, through systematic coding procedures, can effectively extract core categories from a large amount of textual data and construct the logical relationships between them, making it suitable for explaining the internal driving factors of complex social phenomena like online collective action (Dai and Zhu, 2024). After the coding is completed, the theoretical model derived from grounded theory is further compared and verified with relevant research conclusions to enhance the reliability of the theory. The specific methods of grounded theory research are shown in Figure 1.

### 2.4 Data sources

In terms of data collection, the document collection method was adopted. The documents mainly consist of journal articles from CNKI and news reports on social media platforms. Data was sourced by organizing cases of online collective action in recent years, searching CNKI for various related literature (including theses, newspapers), and using websites like Baidu and Weibo to search for related news reports, topic-related microblogs, and event comments. To ensure the usability, objectivity, and authority of the data, we actively screened relevant literature from CNKI that ranked high in downloads and citations. When searching for news reports, we ensured the reputable authority of the media organizations, selecting reports with high discussion and forwarding rates. When capturing comments, we selected popular comments with a high number of likes and replies to ensure the content was diverse and representative. In addition, “online collective action” or “online collective action” (in Chinese) were used as keywords to search for related case-study papers on CNKI as a data supplement. To ensure data richness and the possibility of triangulation, this study collected over 300,000 words of textual data, which were imported into NVivo 14 software for unified management and analysis. Research case information is shown in Table 1.

**Table 1:** Basic information of research case samples.

**Case ID**	**Case name**	**Type**	**Main platform**	**Time**	**Analyzed text volume (words)**
C01	Sun Haiyang child search	Social welfare	Weibo, Douyin	2021–2022	approx.25,000
C02	Zhang Xiaoquan kitchen knife	Livelihood rights	Weibo, Zhihu	2022	approx.20,000
C03	A certain fan group “hitting the ranks”	Culture and entertainment	Weibo	2019	approx.18,000
⋯	⋯	⋯	⋯	⋯	⋯
C16	“Di Ba” expedition	National sentiment	Tieba, Weibo	2020	approx.30,000
Total	16 Cases				>300,000

### 2.5 Researcher positionality and analytical rigor

This study employs constructivist grounded theory as its methodological foundation, emphasizing the interaction and co-construction of meaning between the researcher and the data. In this process, the researcher's positionality, background, and reflective practice significantly influence theory construction. The core research team for this study comprises individuals with backgrounds in sociology and management, providing an interdisciplinary theoretical perspective and training in qualitative research. Throughout the research process, the researchers maintained a conscious reflection on their own interpretive stance, explicitly acknowledging that this study is guided by a constructivist epistemology—viewing social phenomena and meaning as continuously constructed through interaction, rather than as objective realities independent of the observer.

To enhance the transparency and theoretical representativeness of case selection, this study adopted a combined approach of theoretical sampling and maximum variation strategy, selecting 16 online collective action events. Cases were chosen based on the following principles:

Typicality: the cases had high visibility and public influence on mainstream platforms such as Weibo and Tieba.

Diversity: cases covered various types, including social welfare, livelihood rights, cultural entertainment, and national sentiment, to capture the multidimensional aspects of online collective action.

Theoretical relevance: the cases exhibited distinct characteristics in emotional mobilization, identity construction, and action constraints, facilitating the extraction and saturation of theoretical categories.

Through constant comparison and theoretical sampling, the process continued until new cases no longer yielded new categories or relationships, achieving a state of theoretical saturation.

During the coding process, to ensure analytical reliability and consistency, the research team implemented rigorous measures: Two researchers independently performed open coding on a portion of the samples, followed by a consistency check. Theoretical memos were continuously written at various coding stages to document the thought process behind category formation, relationship construction, and theory evolution, enhancing the traceability of the analysis. Beyond social media texts, multiple data sources such as news reports and academic literature were incorporated for triangulation of initial categories. Regular discussions were held with qualitative research experts outside the core team for critical review and revision of the coding framework and theoretical model.

The researchers' disciplinary backgrounds and value positions may influence data interpretation and theory building. To address this, we consciously avoided preconceived judgments during analysis, prioritized allowing categories to emerge naturally from the data, and continually refined interpretive perspectives through team discussions and external feedback, striving to maintain reflexivity, transparency, and rigor throughout the constructive process.

## 3 Category refinement and preliminary model construction

### 3.1 Open coding in grounded theory

Open coding is a key step used for the initial analysis and summarization of collected raw data. This process mainly involves intensive data inspection and analysis to reveal key information and features in the raw data. Open coding is characterized by openness and flexibility; this step relies on the researcher's subjective assessment and experience. During the coding process, researchers must set aside personal biases, conduct in-depth observation, recording, and analysis of the data, and constantly adjust and correct their understanding of the data. This allows researchers to transform raw data into a more basic, comparable form, providing a foundation for subsequent theory construction.

In the open coding stage, sentences in the raw data are analyzed sentence by sentence and paragraph by paragraph, extracting keywords, phrases, or short sentences, and classifying them into different themes or categories. This process is one of continuous iteration and optimization, during which the coding results must be repeatedly reviewed and adjusted to ensure their accuracy and reliability. The purpose of open coding is to discover hidden patterns, trends, and relationships in the data, providing empirical support for later theory construction. This enables researchers to gradually discover the basic concepts and categories of the research object, laying a foundation for further theoretical analysis. Through continuous comparison of the data, a total of 288 concepts were obtained, and the open coding results were classified according to the source of the coding data. Some of the open coding results are shown in Table 2.

**Table 2:** Open-ended coding example.

**Case ID**	**Brief description**	**Original statement**	**Conceptualization**
C01 Sun Haiyang child search	Public welfare empathy and sustained public opinion supervision	A tenacious, responsible, and wise father, leading the family from chaos to harmony.	Sun Haiyang's perseverance differs from the impetuous style of modern society a2
C02 Zhang Xiaoquan kitchen knife	Product quality doubts and corporate trust crisis	What angered everyone was not the knife, but the arrogant attitude of the company's management toward consumers.	Arrogant attitude triggers negative public opinion a9
C03 A certain fan group “Hitting the ranks”	Highly organized fan collective action	The management team is the leader of the community, and the rules and regulations within the group are generally formulated by them.	Community leaders bear more responsibility and have more power a51
C04 Dong Yuhui incident	Fan rights defense action centered on “de-personalization”	Because trust begins with the understanding of emotions and feelings, or in other words, empathy.	Need for empathy between streamer and consumer a74
C05 Li Jiaqi & Florasis	Identity Crisis Triggered by Streamer's Remarks	Mocking and simultaneously pitying old domestic brands for their “poverty”.	Consumers like the unique frugal characteristics of old domestic goods a4
C06 Pang Donglai defends employee	Corporate responsibility triggers positive collective praise	Once the investigation results were released, netizens praised it, “This kind of company is truly people-oriented”	People-oriented, good-attitude solutions win praise a14
C07 “Ice cream price assassins”	Collective mockery and criticism of unreasonable pricing	netizens expressed that they were not targeting just one ice cream, but Zhong Xue Gao's arrogant attitude.	Consumers dissatisfied with the brand's arrogant attitude a12
C08 Yu Wenliang goes viral	Collective identification with and self-expression of “ordinariness”	We can see Yu Wenliang doesn't use many filters, doesn't make everyone anxious, he is very real.	Yu Wenliang is liked by the audience for his authenticity a33
C09 BMW mini ice cream incident	Differential treatment stirs national sentiment	Is it because wealth makes one arrogant, or is it arrogance and ignorance at play?	Arrogance and ignorance cause dissatisfaction a1
C10 Xiaoguo talk show	Offending mainstream social values triggers denunciation	Talk shows shouldn't get laughs by breaking mainstream values	Talk shows should not break mainstream values a6
C11 Panda YaYa incident	Animal protection sentiments and transnational public pressure	The responsibility for the mistreatment of the panda in the US lies with the “CAZG and the Memphis Zoo genetic breeding lab”	Some netizens are concerned about the accountability for the incident a52
C12 Community WeChat groups respond to COVID	Community self-organization and mutual aid under crisis	These feelings and emotions infect each other through homeowners' verbal interactions, promoting active participation in event discussion and action.	Discourse leads to emotional contagion among homeowner groups a82
C13 Production crew cat abuse incident	Collective accountability driven by animal protection awareness	These netizens generally believe that Yu Zheng harmed an innocent life under the guise of art.	More negative personal evaluations a3
C14 Fans' “over 100 million” retweets	Fan “Brushin” competition behind data fabrication	For younger groups, the plasticity and assimilation power are very strong	Celebrities themselves have a large influence on young groups a69
C15 Defective vaccine incident	A public trust crisis concerning public safety	I really can't imagine, how much truth have certain interest groups concealed?	Concealing the truth angers people a26
C16 “Di Ba” expedition incident	Organized expression of online nationalism	During the dissemination of public issues, netizens compete to speak, forming a competition of diverse discourses.	Topics that arouse emotional resonance among netizens receive high attention a86

### 3.2 Relational coding

In grounded theory, relational coding is an important step. Its main task is to discover and establish different relationships between conceptual categories to represent the organic connections between various parts of the data. These connections can be causal, temporal, similarity-based, etc.

The relational coding process involves in-depth analysis and integration of existing concepts. Through multiple reflections and analyses of the relationships between concepts, more abstract categories are integrated, and the properties and dimensions of the relevant categories are determined. In this process, the researcher must maintain a high degree of theoretical sensitivity, optimizing and enriching the theoretical content by repeatedly comparing data and theory, and finally classifying the formed concepts to obtain categories. In this part, concepts with similar meanings are preliminarily clustered to form 52 sub-categories, and some of the associated coding processes are shown in Table 3.

**Table 3:** Relational coding examples.

**Concept**	**Sub-category**
Arrogance and ignorance cause dissatisfaction a1	Personality traits b1
Talk shows should not break mainstream values a6	Value orientation b2
Arrogant attitude triggers negative public opinion a9	Arrogant attitude b3
Streamer's confrontational interpretation hurts consumer feelings a13	Handling method b4
Dissatisfaction with concealing the truth a25	Concealing truth b5
Brand's passive handling attitude causes dissatisfaction a28	Passive handling b6
Yu Wenliang is liked by the audience for his authenticity a33	Genuine expression b7
Enterprise's practices are honest, winning praise a38	Active and responsible b8
The vast majority of ice creams are sold at high prices through online marketing a44	Marketing strategy b9
Enterprises need the courage to admit mistakes a49	Attitude toward admitting mistakes b10
The incident reflects Xiaoguo's lack of content control a50	Responsibility bearing b11
Streamers need a good reputation a53	Past reputation b12
Bullying under the pretext of defending an idol a58	Negative impact b13
Public's associations from the event make emotions more intense a64	Generating associations b14
…	…
Poor living conditions for giant panda YaYa lead to poor physical condition a224	Factual basis b41
The Internet endows memes with new connotations, providing conditions a228	Communication medium b42
Streamers play an important role in the sales process a241	Work content b43
Enterprises have a high degree of transparency to consumers a253	Information disclosure b44
Abiding by the law is a basic principle a256	Legal bottom line b45
Social development and individual self-confidence increase a257	Social development b46
Enterprises need to undertake social responsibility to be respected and trusted a258	Social responsibility b47
Lack of price transparency causes consumer dissatisfaction a265	Management defect b48
Non-compliance with rules will be punished a270	Strict discipline b49
Practitioners believe some peers lack artistic ethics a278	Professional bottom line b50
Practitioners believe the talk show industry also needs time to mature a285	Industry status b51
Contempt for life touches the moral bottom line a287	Moral bottom line b52

### 3.3 Axial coding

**Figure 2:**
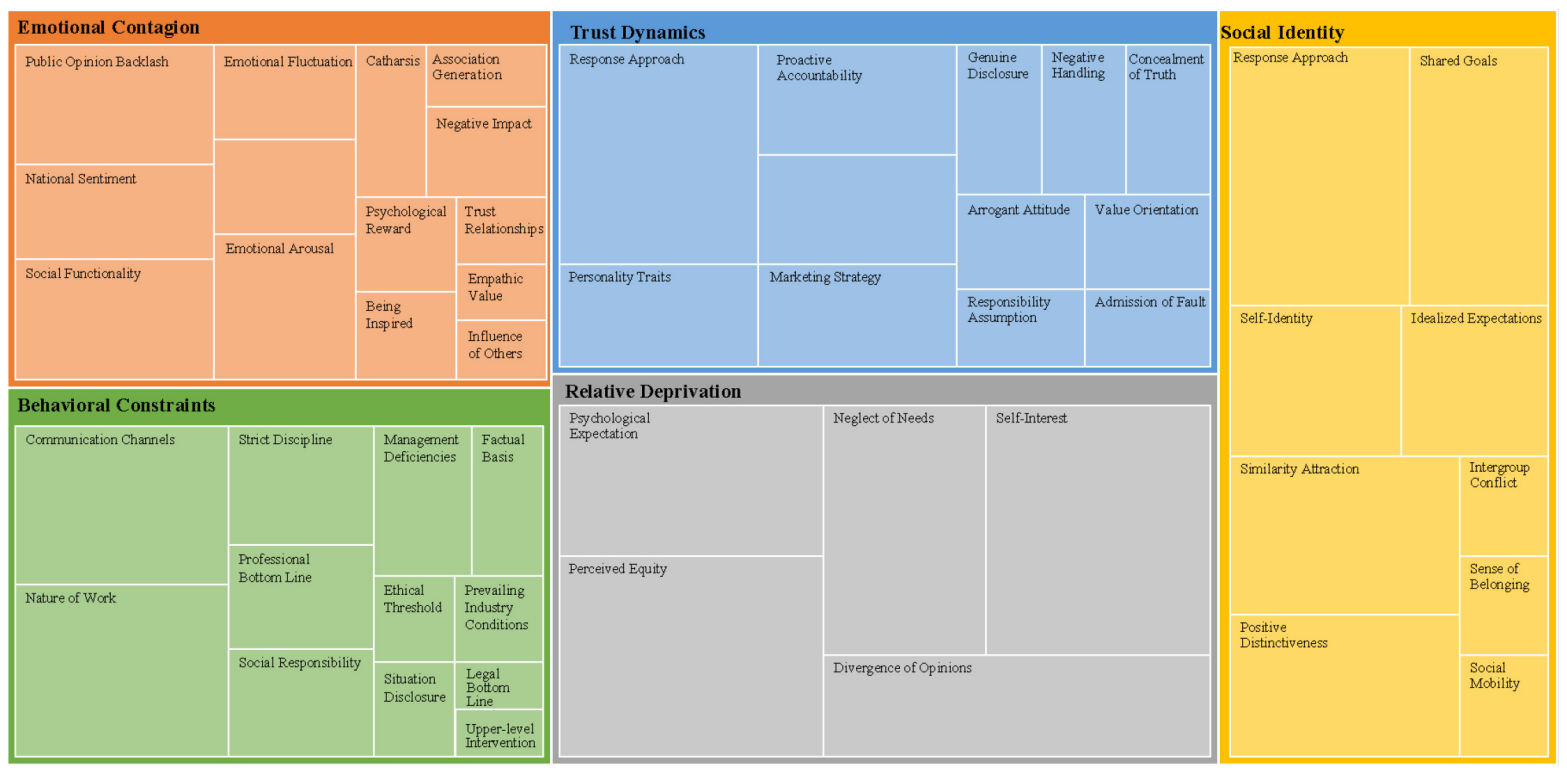
Axial coding tree hierarchy diagram.

In this part, the model “Inclusion Relationship—Influencing Factors—Action Methods—Final Results” is used to establish the model for each category, and from this, the main categories are selected, i.e., the categories most relevant to the influencing factors of online collective action incidents. In this step, we first review all the concepts and categories obtained during the open coding and relational coding stages. Second, these concepts and categories are organized, classified, and summarized to discover the associations and hierarchical structures between them. Then, among the organized concepts and categories, key concepts that appear frequently, hold a central position, or can connect other concepts are identified. Finally, similar or related concepts are clustered to form one or more concept groups, ensuring that each concept group can reflect a relatively independent and complete theme or domain, and that there is a certain logical connection. After organization, five main categories were finally formed: emotional contagion, trust dynamics, relative deprivation, social identity, and action constraints. After visualizing the final results, the tree structure diagram of the coding process is shown in Figure 2, and the main axis coding process is shown in Table 4.

**Table 4:** Main categories and their corresponding sub-categories.

**Main category**	**Connotation of main category**	**Sub-category trust dynamics**
Trust changes	In online collective action, the public's trust in individuals, organizations, or institutions undergoes dynamic changes due to factors such as personality traits, value orientations, attitudes, behaviors, problem-handling methods, and past reputations. This change directly affects the public's willingness to participate and their behavioral choices, including the processes of trust establishment, destruction, and repair.	Personality Traits b1, Value Orientation b2, Arrogant Attitude b3, Handling Method b4, Concealing Truth b5, Passive Handling b6, Genuine Expression b7, Active & Responsible b8, Marketing Strategy b9, Attitude toward Admitting Mistakes b10, Responsibility Bearing b11, Past Reputation b12
Emotional contagion	In online collective action, individual emotions spread rapidly and are amplified through social interaction, forming a group emotional phenomenon. This process involves psychological mechanisms such as empathy, emotional resonance, and emotional arousal, and is moderated by factors like the influence of others, trust relationships, and national sentiment. Emotional contagion can trigger collective action but may also lead to negative impacts or public opinion backlash.	Negative Impact b13, Generating Associations b14, Influence of Others b15, Trust Relationship b16, Empathic Value b17, Feeling Encouraged b18, Emotional Arousal b19, Emotional Resonance b20, Emotional Change b21, Emotional Venting b22, National Sentiment b23, Social Function b24, Public Opinion Backlash b25
Relative deprivation	An individual or group perceives themselves to be in a disadvantaged position regarding treatment, benefits, or rights through social comparison, thereby generating dissatisfaction and resentment. This sense of deprivation stems from the gap between psychological expectations and reality, the lack of equal treatment, needs being ignored, differences of opinion, or harm to self-interest, which in turn drives participation in collective action to seek change.	Equal Treatment b26, Psychological Expectation b27, Ignoring Needs b28, Difference of Opinion b29, Self-Interest b30
Social identity	An individual gains a sense of belonging and self-worth by identifying with a specific group or ideology. This identification is based on factors such as common pursuits, ideal expectations, and similarity attraction, and it reinforces group boundaries through positive differentiation. Social identity motivates individuals to participate in collective action to maintain group interests or respond to group conflicts, while simultaneously enhancing self-identity and collective identity.	Common Pursuit b31, Status Mobility b32, Sense of Belonging b33, Ideal Expectation b34, Positive Differentiation b35, Similarity Attraction b36, Group Conflict b37, Self-Identity b38, Collective Identity b39
Action constraints	Refers to the various internal and external factors that limit and regulate individual or group actions in online collective action. These constraints include upper-level intervention, legal and moral bottom lines, factual basis, information disclosure, communication medium characteristics, work content, social responsibility, management defects, disciplinary requirements, professional norms, industry status, and social development needs. Action constraints shape the form, scope, and effectiveness of collective action.	Upper-level Intervention b40, Factual Basis b41, Communication Medium b42, Work Content b43, Information Disclosure b44, Legal Bottom Line b45, Social Development b46, Social Responsibility b47, Management Defect b48, Strict Discipline b49, Professional Bottom Line b50, Industry Status b51, Moral Bottom Line b52

### 3.4 Theoretical saturation test

The final stage of grounded theory research is the theoretical saturation test, which aims to verify the completeness and depth of the constructed theory with new samples and to ensure that the core categories and their internal relationships have stabilized. This study randomly selected three cases from the original 16 research cases for saturation testing.

Case A (A certain celebrity fan's “over 100 million” retweets incident): The fan group forms community connections based on common hobbies-Social Identity. Driven by emotions for the idol, they establish a common goal-Emotional Contagion. They achieve over 100 million retweets through a hierarchical, organized, and efficient operational mechanism–Action Constraints.

Case B (Zhang Xiaoquan kitchen knife incident): The brand failed to effectively respond to consumers' core demands regarding product quality-Relative Deprivation. Its passive response strategy of shirking responsibility triggered strong consumer dissatisfaction-Trust Dynamics. This was compounded by the brand executive's past inappropriate remarks touching national sentiments-Emotional Contagion, ultimately leading to large-scale boycott behavior.

Case C (“Di Ba” expedition incident): Community members perceived injustice due to conflicts with other groups-Relative Deprivation. Under the call and organization of the Tieba administrators, i.e., opinion leaders-Action Constraints, they implemented online collective action in the form of “forum bombing” (“baoba”). This community had also initiated cross-border online collective actions based on consensus, such as safeguarding the national stance-Social Identity.

**Figure 3:**
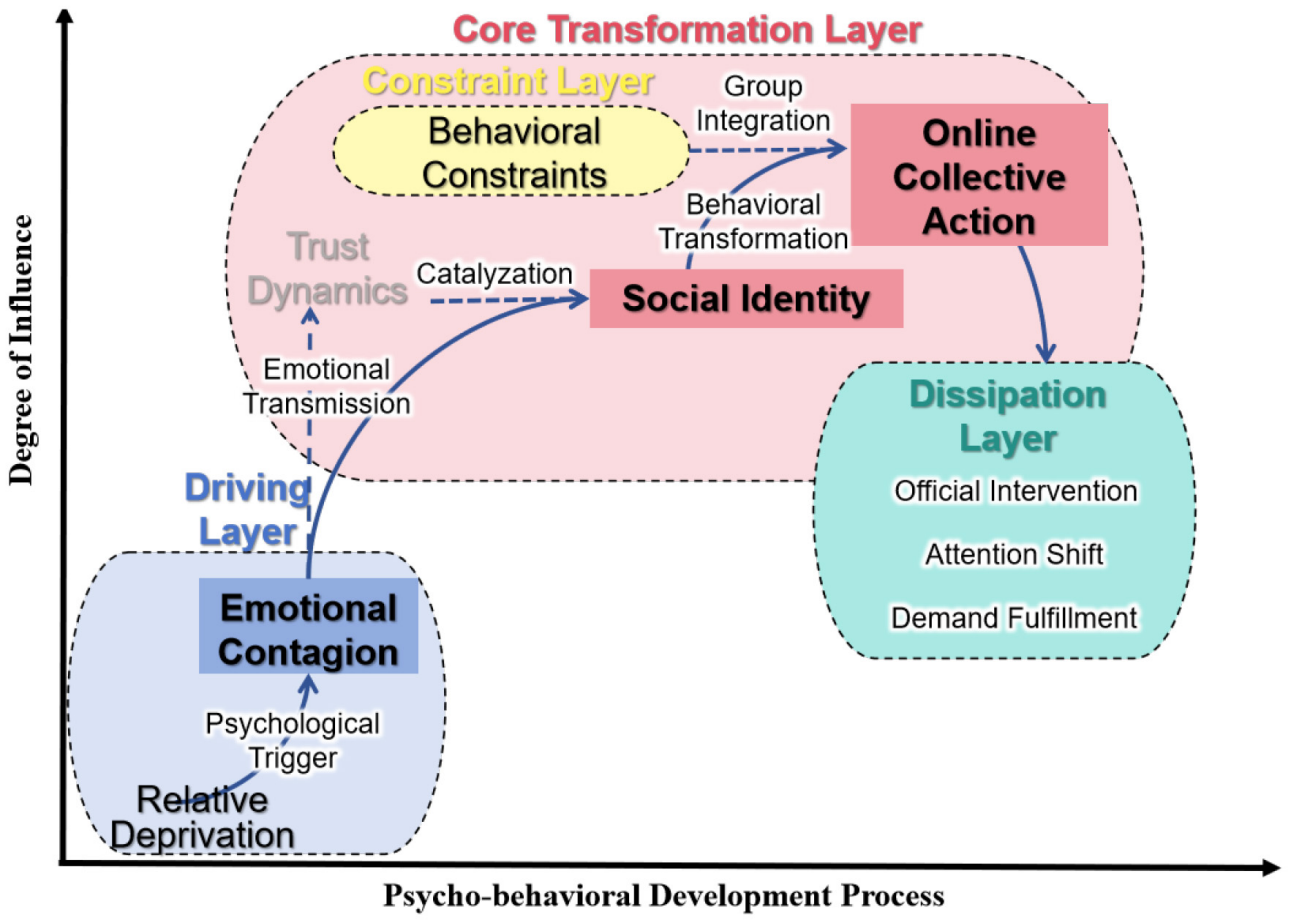
Online collective action model diagram.

The analysis of the three test cases above did not reveal any new heterogeneous information regarding the core categories and their relational pathways, and the quantity has met the saturation requirement. Based on this, it can be determined that the theoretical model has reached a state of saturation. Finally, based on the logical connections between the core categories, the following theoretical model was constructed, as shown in Figure 3.

As shown in Figure 3, the core storyline can be described as: Online collective action is a process where negative emotions, triggered by a sense of relative deprivation or a trust crisis, are rapidly spread and amplified through an efficient emotional contagion mechanism within a group possessing high social identity, and under the catalysis of specific action constraint conditions, it ultimately transforms into online or even offline collective action.

## 4 Model elucidation

### 4.1 Emotion is the core driving thread throughout

In an uncertain environment, emotion profoundly influences an individual's interpretation of the essence of an event. It is both an intrinsic component of the event's repercussions and a key factor that shapes and guides the direction of action, oriented by stable emotional bonds. Furthermore, emotion constitutes the core cohesive force for maintaining a community; without the infusion of emotion and feeling, social movements cannot be discussed (Shi and Tang, 2023). Therefore, the role of emotion as a group adhesive is irreplaceable. Emotion, as a prerequisite for action, will quickly spread and unify within a group, forming a powerful group emotional force, which is emotional contagion. This spread and diffused emotional force is stimulated and released under the guidance of leaders, thus promoting the collective to take unified action (Bon, 2000).

Compared to real life, the cost of expressing emotions in the digital realm is lower. Simultaneously, in the process of constructing self-organized behavior, extreme views and inflammatory rhetoric are often more easily recognized and responded to. This illusory sense of power further induces group members to adopt more extreme stances (Huang and Liu, 2023). Through the expression of emotion, collective mobilization at the emotional level is effectively promoted, ultimately giving rise to online collective action. This situation has already become a new feature of the dynamic evolution of public opinion in the digital age. The intensity and speed of emotional contagion are significantly amplified in the online environment. Neuroscience research further supports this mechanism (Zhu et al., 2025), finding that positive emotional contagion is achieved through facial expression synchronization and inter-brain synchronization via the mirror neuron system, triggering automatic imitation even in interactions between strangers. This provides an explanation for emotional transmission in cyberspace, where non-verbal cues are absent. Furthermore, workplace research found that negative gossip weakens the self and leads to counterproductive behavior through emotional contagion (Abdelghani et al., 2025), highlighting the negative impact of emotional contagion in contexts of broken trust (Abdelghani et al., 2025).

Due to the lack of an institutionalized and organized background for online collective action in China, the role of participants' emotions in the mobilization of online collective action is prominent (Xu, 2021). Emotion not only plays a key driving role in online collective action but can also be said to be one of the necessary prerequisites. Emotional expression in cyberspace has a strong amplification effect (Wu et al., 2024; Bai, 2022) and contagiousness (Xu et al., 2024). In the era of internet popularization, once a certain emotion spreads widely on the internet, it will quickly trigger collective resonance. Emotion, as a special and important function of humans, powerfully promotes the occurrence of online collective action.

The “emotional drive” model constructed in this study stands in stark contrast to traditional collective action theories based on rational calculation. Classical theories emphasize the rational decision-making process of individuals based on cost-benefit analysis, resource integration, and organizational mobilization, with the core assumption being the strategic participation of the “rational actor” in collective action. However, in the online collective action of the digital age, it is often emotion, rather than rational calculation, that becomes the core driving force for the initiation and spread of action. Emotion not only lowers the psychological threshold for participation but also achieves large-scale, non-organizational mobilization through instant and widespread social interaction. This model reveals that emotional contagion can spontaneously form the motivational basis for collective action in the absence of clear organizational leaders or material incentives, challenging the “rationality-dominated” premise of traditional theories and highlighting the emotional turn of collective action in the digital age.

The “emotion-driven” model constructed in this study engages in a profound theoretical dialogue with the dominant rationalist paradigm in collective action research. Classical theories, exemplified by the “Logic of Collective Action” (Olson, 1965), are predicated on the core assumption that rational individuals participate in collective action based on cost-benefit calculations, with a primary focus on analyzing solutions to the “free-rider” dilemma and selective incentives. Subsequently developed theories, such as Resource Mobilization Theory and the Political Process Model (McCarthy and Zald, 1977), while expanding their perspectives to include organizations, resources, and political opportunity structures, still treat emotion as a subordinate variable that needs to be managed, controlled, or strategically framed. Their core remains an action logic dominated by “instrumental rationality”. In contrast, this model reveals that in the digital era's online collective actions, emotion is not an interference to rational decision-making nor an inert resource awaiting “mobilization.” Instead, it is a spontaneous, contagious, and self-evolving driving core. Emotional contagion can achieve low-threshold, nonlinear rapid mobilization even in the absence of formal organization and material incentives. The dynamic interaction between emotion, identity, and technological constraints constitutes the key to understanding how actions emerge, evolve, and even dissolve. Therefore, the contribution of this model lies not in negating rational or structural factors, but in shifting emotion from the periphery to the center of analysis and systematically integrating it with meso- and macro-level factors. It thus provides an alternative analytical framework that better aligns with the context and dynamic essence of highly mediated, decentralized, and emotion-saturated contemporary online collective action.

### 4.2 A sense of relative deprivation as the root-level driving factor

Normally, the generation of a sense of relative deprivation is not directly related to the increase or decrease of one's own interests, but stems from the choice of a reference object. For example, some individuals may have been satisfied with their own situation in their original living environment, but due to exposure to groups and economic conditions completely different from their native environment, they change the reference for judging their own value, and a sense of relative deprivation arises from this (Hu, 2024). Besides, a sense of relative deprivation is not only an initial driver of collective action but may also lead to social alienation and radicalization. Research across multiple European countries found that personal economic relative deprivation and group injustice jointly predict social alienation, and anti-mainstream identity reinforces alienation through a sequential mediation of group injustice and a sense of humiliation (Melita et al., 2025), which explains the formation basis of “adversarial identity” in cyberspace.

With the vigorous rise of new media, individuals on the internet can more intuitively see the colorfulness in others' lives. When such exciting life fragments continuously emerge on internet platforms, they actually construct an illusion that seems ubiquitous and easily attainable. People gradually realize that although these life scenes are close at hand, they are in fact difficult to reach due to various real-world constraints. This gap between the ideal and reality often stimulates the breeding of frustration, anger, and dissatisfaction (Yang and Xue, 2024), constituting a rather unique psychological phenomenon in the new media era. Relative deprivation stems from social comparison, which is especially easily triggered by exposure to digital media. Research on China's poverty alleviation relocation shows that different relocation methods may increase immigrants' economic, social, and emotional deprivation, thereby affecting psychological adaptation (Li M. et al., 2025), reflecting the complexity of relative deprivation under policy intervention. Research further differentiates the impact of emotional abuse and neglect on aggressive behavior (Xie et al., 2025), where relative deprivation is a key mediator through which emotional abuse leads to aggression (Xie et al., 2025), indicating the bridging role of deprivation in transforming negative emotions into action.

Obviously, the core of a sense of relative deprivation lies in a sense of unfairness. In the process of advancing Not In My Back Yard (NIMBY) projects, although they can promote the overall welfare of society, they inevitably impose certain negative externalities on the nearby resident groups (Chen and Qian, 2024). In this situation, the significant asymmetry between the benefits perceived by the individual residents and the costs they bear directly triggers their sense of relative deprivation. Subsequently, this feeling may drive residents to freely express dissatisfaction online, further triggering emotional contagion. They may also take collective action, attempting to “correct” their perceived injustice and deprivation.

### 4.3 The behavioral path of emotional contagion, social identity, and the formation of online collective action

In cyberspace, individuals tend to seek a sense of belonging and identity by joining specific communities or organizations. When a certain community or organization is threatened or challenged externally, its members often show strong unity and willingness to act to maintain their social identity. The process of emotional contagion is not only the transmission and sharing of emotions between individuals but also an important way for social identity to form. When individuals resonate emotionally with others, they are more likely to see themselves as members of a certain social group, thereby enhancing their sense of identity and belonging to that group. In the occurrence of online “mass rage,” emotional contagion also plays a key role: netizens, through common emotional experiences such as moral and value judgments, generate emotional resonance, and then develop empathy for the subject involved. This empathy comes from common emotional experiences, value judgments, etc., making it easier for netizens to stand on the same side and show sympathy and support for the subject (Cheng and Xu, 2022).

The interactive effect of social identity and emotional contagion is particularly significant in online collective action (Kong et al., 2025). EEG experiments have demonstrated that identity information top-down modulates racial emotional contagion, indicating that a sense of group belonging can amplify emotional resonance. This supports the emotional reinforcement of the “us” vs. “them” boundary in online action. In sports teams, it was found that team identity enhances teamwork through the transactive memory system, and adaptive responses differ significantly under time pressure (Özlü et al., 2025), providing an analogy for the formation and evolution of group cohesion in online collective action. Social exclusion increases aggressive behavior through relative deprivation, and upward social comparison strengthens this pathway, revealing the synergistic mobilization mechanism of identity threat and emotional contagion (Yu et al., 2025).

In the context of emergencies, individuals tend to actively move closer to the communities they identify with, forming an aggregation phenomenon. The strength of their social identity is directly related to the frequency of emotional contagion, which in turn affects the proactiveness of emotional expression. This type of behavior reflects a herd mentality of following and imitating. Especially when the group size expands, the tendency for conforming behavior increases significantly (Song and Chang, 2021), meaning that an increase in the number of group members will exacerbate this phenomenon.

The formation pathway of online collective action is deeply influenced by platform characteristics and action support. Social media forms a “shared reality” by documenting individual experiences, building communities, and shaping norms, which is particularly crucial in online environments lacking organizational structure (Greijdanus et al., 2020). However, platforms also enhance the visibility of actions, making them susceptible to governance intervention, highlighting the double-edged sword effect of online action. Furthermore, the current prominence of online collective action is inextricably linked to the technical characteristics of social media platforms. First, algorithmic recommendation mechanisms, through personalized content distribution, reinforce the “information cocoon” and “echo chamber” effects, allowing similar emotions to be continuously strengthened and circulated within groups, accelerating emotional homogenization and polarization. Second, instant interaction and low-barrier dissemination make emotional expression and contagion almost zero-latency and zero-cost; emotions can achieve exponential diffusion in a very short time. In addition, multimedia and symbolic expression (e.g., memes, emojis) enhance the visual impact and contagious power of emotions, making abstract emotions concrete and simplifying complex feelings, which more easily triggers widespread resonance. These technical characteristics work together to not only amplify the intensity and speed of emotional contagion but also to strengthen the construction process of social identity–individuals, in emotional resonance, quickly form a “we” group boundary, thus achieving efficient emotional mobilization in the absence of a traditional organizational structure.

### 4.4 Contextual analysis of failure to translate into collective action

Although the “emotional driving force” model constructed in this study emphasizes the core role of emotion in online collective action, emotional mobilization does not inevitably lead to collective action. In reality, numerous contexts exist where significant emotional arousal fails to culminate in sustained or effective collective action. These “incomplete” emotional mobilization processes precisely reveal the boundary conditions and contextual dependency of the proposed model.

Firstly, insufficient intensity or sustainability of emotion may confine emotional mobilization to the level of individual expression. For instance, in certain transient online hotspots (e.g., controversial remarks by a celebrity), while emotions are ignited, the lack of continuous emotional fuel and narrative depth leads to the rapid dissipation of emotional energy, preventing the formation of collective identity and coordinated action.

Secondly, the absence or fragmentation of social identity undermines the organizational foundation for emotional mobilization. If the emotional subjects fail to develop a clear “we” identity, or if internal group identification is divided, even intense emotion struggles to translate into unified collective action. For example, in discussions on some public issues, despite strong netizen sentiment, polarization of stances and factional opposition prevent the emergence of a unified course of action.

Thirdly, the strengthening of action constraints can directly obstruct the translation of emotion into action. Platform measures such as content moderation, traffic limiting, or account suspension, or the materialization of offline legal risks, significantly increase the cost of action and inhibit the formation of collective action. For instance, regarding some sensitive social topics, despite accumulating online sentiment, stringent platform controls and a shrinking space for discourse prevent emotion from openly evolving into organized action. Furthermore, the absence of leaders or insufficient organizational resources is another crucial reason for the failure of emotional mobilization. While emotion can spread spontaneously, a lack of guidance from opinion leaders, support from organizational structures, or resource coordination mechanisms hampers the conversion of emotion into sustainable collective action. For example, in certain sudden public opinion events, high netizen emotion coupled with a lack of coordinating bodies results in action remaining at the level of verbal expression, failing to achieve online-offline synergy.

Finally, the disconnect between emotional expression and action goals may also lead to mobilization failure. If emotional expression is not coupled with a clear action framework or feasible solutions, emotion may dissipate as mere venting rather than channeling toward concrete action. For instance, in some complex public crisis events, while public emotion is strong, the complexity of the problem, ambiguity of responsible entities, or unclear resolution paths prevent emotion from transforming into targeted collective action.

These contexts indicate that the emotional driving force model is not a deterministic, linear logic but is moderated by multiple conditions such as emotional quality, identity structure, constraint intensity, and organizational resources. Future research could deepen the understanding of the boundaries of emotional mobilization by comparing cases or tracing the process of emotional dissipation, thereby enhancing the model's contextual sensitivity and explanatory power.

## 5 Conclusions and implications

### 5.1 Recognizing the central role of the affective dimension in public governance

This study finds that emotion serves as the central driving thread of online collective action. This insight highlights that affect constitutes a dimension that cannot be overlooked in public governance and policy communication (Ma and Chai, 2024). This aligns with research on emotional governance, which emphasizes the critical importance of interventions designed around the mechanisms of emotional contagion. For instance, the sense of relative deprivation influences psychology and behavior through the perception of social fairness (Zhuang et al., 2025). This suggests that incorporating responses to fairness concerns in public communication may help channel potential collective sentiments. Evacuation simulations indicate that dynamic guidance in emergency scenarios can accelerate emotional stabilization (Wang et al., 2025), providing a reference for real-time emotional management during online public opinion crises (Wang X., 2023). Therefore, the model implies that policy narratives aimed at channeling emotions or building consensus may need to move beyond mere information dissemination, striving instead to construct meaningful frameworks capable of eliciting emotional resonance and integrating diverse value claims.

Concurrently, policy communication faces a fundamental challenge. In today's modern information era, which prioritizes efficiency and speed, individuals inevitably and frequently encounter a deluge of information in daily life. Policy information is often submerged within irrelevant content. This situation makes it difficult for governments to ensure policy messages receive sufficient attention during dissemination (Ji and Xiao, 2023). Information characterized by high exposure, ease of access, and intuitiveness often stands out, attracting and occupying more attentional resources. Furthermore, the efficiency of emotional contagion is linked to attention concentration. This suggests that in a media environment overloaded with information, the effectiveness of public communication may depend not only on the content itself but also on its ability to effectively reach and sustain the emotional attention of the public within a complex communication ecosystem.

### 5.2 Rebuilding trust through information transparency and empathetic communication

Emerging media, represented by the Internet, mobile phones, and social media, play an important role in the formation of political trust. The popularization of emerging media has broken down the barriers of time and space in information dissemination, making the acquisition of political information more convenient and diverse. The impact of emerging media on political trust is complex and varied. It may enhance political trust by providing rich political information, or it may weaken political trust due to the fragmentation and emotionalization of information dissemination (Ji and Xiao, 2023). From a criminological perspective, behavioral risks triggered by relative deprivation stem from a lack of justice, further confirming the importance of transparent and fair policy processes in alleviating feelings of deprivation (Itskovich, 2025). A transparent and trust-based information ecology can alleviate relative deprivation and trust crises. Algorithmic control leads to job burnout through relative deprivation, with digital technology identity playing a moderating role, suggesting platforms need to enhance algorithmic transparency to maintain trust (Zhang C. et al., 2025). A meta-analysis found that leader bottom-line mentality is positively correlated with relative deprivation and moral disengagement, indicating the criticality of organizational justice for trust-building (Luan et al., 2025).

The model reveals that “trust dynamics” act as a key pivot shifting accumulated emotion into willingness for action (Zhang, 2024). When a negative event triggers initial distrust, the manner in which information is handled determines whether trust collapses or is repaired. Therefore, establishing transparent, timely, and empathetic communication channels is central to interrupting a vicious cycle of distrust. For responsible entities, this implies: First, during a crisis, prioritizing the transparency and continuous updating of key information to fill the “information vacuum” and curb the intensification of relative deprivation and anger fueled by rumor proliferation. Second, the tone of communication is crucial; avoiding defensive, bureaucratic, or arrogant lecturing in favor of demonstrating listening, empathy, and a responsible demeanor. Third, establishing and publicizing a closed loop for feedback and accountability, allowing the public to see that opinions are heard and issues are addressed. The essence of these practices is to treat communication itself as the core intervention for rebuilding trust.

### 5.3 Guiding community identity toward rationality and constructiveness

In current online communities, the density of users' shared information attention networks is low, showing a trend toward stratification and “silos.” Guiding identity requires consideration of group differences and identity dynamics. Demonstrating superiority through moral behavior rather than bullying helps maintain healthy self-efficacy and social support (Saripanidis et al., 2025), offering a pathway for positive identity guidance. Research in South Korea found that intergenerational differences in relative deprivation and social comparison affect mental health (Shin et al., 2025), indicating that policies need to design identity guidance strategies tailored to different age groups. This study reveals a reinforcing cycle between social identity and emotional contagion. This provides a line of thinking for guiding online community culture.

Based on this reinforcing cycle, the model implies that a completely homogenized information environment may accelerate emotional polarization and identity closure within groups (Yang F. et al., 2023). Therefore, within platform ecosystems and governance, exploring how to strategically introduce constructive heterogeneous viewpoints may help increase the elasticity of group identity and moderate the momentum toward extremism.

Simultaneously, research finds that key nodes within communities (such as opinion leaders) play a significant role in setting discussion frameworks and shaping emotional atmospheres. The “trust authority” of community leaders stems from their capacity for interpretation and judgment (Ma, 2024). This suggests that identifying and supporting key users who can promote rational discussion and emotional regulation may leverage their positive demonstration effect, which can diffuse through social networks. Research indicates that emotion regulation strategies like cognitive reappraisal are contagious within groups (Pinus et al., 2025), offering a potential, endogenous pathway for achieving a positive shift in group emotions by empowering key nodes.

Finally, from a longer-term perspective, understanding the affective foundations of social identity also points to the importance of digital literacy education. Phenomena such as “online flaming groups” indicate that deviant behaviors are underpinned by deep-seated identity and emotional needs (Yang and Zeng, 2024). Therefore, cultivating individuals' abilities for critical reflection under group pressure, emotion management, and responsible expression can help lay the micro-foundation for fostering a rational and healthy online community culture.

## 6 Research limitations and future directions

This study, situated within the Chinese social media context, employs grounded theory to construct a model of online collective action with “emotional driving force” as its core motivator. While the model demonstrates strong explanatory power within the selected cases, its application remains context-dependent and bounded theoretically. Future research could test and extend it within broader media and political-cultural systems.

### 6.1 Research limitations

Firstly, all 16 cases selected for this study originate from mainstream Chinese social platforms, where emotional expression, issue nature, and mobilization methods are profoundly shaped by China's unique online ecology and governance environment. Although the cases cover various types such as public welfare, consumption, entertainment, and national sentiment, they do not fully encompass other significant forms of collective action globally, such as election mobilization, social movements, or transnational activism. Secondly, the data primarily consists of publicly available texts. While comprehensiveness was sought, capturing the internal dynamics of non-public communities, offline linkages, and the implicit influence of algorithmic recommendations on emotional diffusion remains challenging. Furthermore, while grounded theory is suitable for theory building, the generalizability of its findings requires further validation through subsequent quantitative research or cross-case comparison.

### 6.2 Discussion on cross-contextual applicability

Within the model, core psychological mechanisms like emotional contagion and social identity likely possess a high degree of cross-cultural universality. For instance, the contagious nature of emotion on digital platforms and the role of group identity in mobilization have been corroborated by social media research in various countries. However, the dimension of action constraints exhibits greater context-specificity. China's platform governance model, content moderation mechanisms, and cyber regulations constitute a unique constraint environment, structurally different from the relatively open speech spaces and community-based content management prevalent in Western contexts. Therefore, in Western or hybrid media systems, action constraints may manifest less as systemic control and more as group norms, platform algorithms, or legal litigation.

Additionally, the construction of relative deprivation is influenced by reference groups and cultural values. In the Chinese context, structural factors like the wealth gap and urban-rural disparities often serve as significant sources of deprivation. In regions with strong individualistic cultures, deprivation might be more closely linked to personal achievement and social mobility expectations. Similarly, the drivers of trust fluctuation vary across political cultures. In China, public trust shifts toward official institutions and corporations are often related to governance efficacy and social responsibility. In some Western countries, trust crises might be more tightly connected to ideological polarization or the elite-mass divide.

To deeply explore the cross-cultural explanatory power and necessary adjustments of this model, future testing in other media systems could follow these paths:

Conduct comparative case studies, selecting collective action events characterized by emotional mobilization on platforms like Facebook, X (formerly Twitter), or Reddit. Apply the model's three core dimensions for analysis, focusing particularly on comparing the differing manifestations of “action constraints.”

Localize the operationalization of key variables. For example, when measuring “relative deprivation,” incorporate locally salient social comparison dimensions such as race or immigrant status. Expand the objects of “trust fluctuation” to include other key local institutions beyond the government, such as NGOs or tech giants.

A further distinction can be made: the elements within the model vary in their degree of universality vs. context-specificity. The interactive mechanisms of emotional contagion and social identity, as fundamental dynamics based on human psychology, possess relatively high cross-cultural universality. In contrast, the specific forms and intensity of action constraints, the sources and targets of relative deprivation, and the key subjects of trust fluctuation represent the most context-dependent parts of the model, deeply reliant on specific political-cultural structures and media governance models.

### 6.3 Future research directions

Future research can advance the cross-cultural testing and theoretical extension of the model in the following directions.

First, systematic cross-cultural comparative research. Select comparable collective action cases from different media and political systems (e.g., Western, East Asian, Middle Eastern). The goal should be not only to compare similarities and differences in emotional mobilization pathways but also to meticulously examine the moderating effects of contextual variables like “action constraints,” thereby clarifying which relationships in the model are universal and which are context-dependent.

Second, investigate the influence of platform mechanisms. Examine how different platform architectures—such as those of X, TikTok, and Facebook—shape processes. Particularly, compare the differential constraining effects of global social media platforms across regions due to local compliance requirements.

Third, employ mixed methods for validation and refinement. Integrate methods like big-data sentiment analysis, social network analysis, and surveys to quantitatively measure and conduct path analysis on the model's emotional contagion pathways, group identity networks, and perceived constraint intensity. This approach can provide quantitative validation and potential refinement of this grounded theory model.

In summary, the model constructed in this study offers theoretical insights for revealing the internal mechanisms of emotional driving forces. However, its completeness and applicability require ongoing validation and refinement through diverse contexts and methodological. It is hoped that this framework can serve as a middle-range theoretical tool for the comparative study of online collective action on a global scale, continually enriching its cultural sensitivity and explanatory flexibility through scholarly dialogue.

## Data Availability

The original contributions presented in the study are included in the article/supplementary material, further inquiries can be directed to the corresponding authors.
